# Expression in Antennae and Reproductive Organs Suggests a Dual Role of an Odorant-Binding Protein in Two Sibling *Helicoverpa* Species

**DOI:** 10.1371/journal.pone.0030040

**Published:** 2012-01-23

**Authors:** Ya-Lan Sun, Ling-Qiao Huang, Paolo Pelosi, Chen-Zhu Wang

**Affiliations:** 1 State Key Laboratory of Integrated Management of Pest Insects and Rodents, Institute of Zoology, Chinese Academy of Sciences, Beijing, China; 2 Department of Biology of Crop Plants, University of Pisa, Pisa, Italy; University of South Florida College of Medicine, United States of America

## Abstract

Odorant-binding proteins (OBPs) mediate both perception and release of semiochemicals in insects. These proteins are the ideal targets for understanding the olfactory code of insects as well as for interfering with their communication system in order to control pest species. The two sibling Lepidopteran species *Helicoverpa armigera* and *H. assulta* are two major agricultural pests. As part of our aim to characterize the OBP repertoire of these two species, here we focus our attention on a member of this family, OBP10, particularly interesting for its expression pattern. The protein is specifically expressed in the antennae of both sexes, being absent from other sensory organs. However, it is highly abundant in seminal fluid, is transferred to females during mating and is eventually found on the surface of fertilised eggs. Among the several different volatile compounds present in reproductive organs, OBP10 binds 1-dodecene, a compound reported as an insect repellent. These results have been verified in both *H. armigera* and *H. assulta* with no apparent differences between the two species. The recombinant OBP10 binds, besides 1-dodecene, some linear alcohols and several aromatic compounds. The structural similarity of OBP10 with OBP1 of the mosquito *Culex quinquefasciatus*, a protein reported to bind an oviposition pheromone, and its affinity with 1-dodecene suggest that OBP10 could be a carrier for oviposition deterrents, favouring spreading of the eggs in these species where cannibalism is active among larvae.

## Introduction

Two sibling Lepidopteran species *Helicoverpa armigera* and *H. assulta* are major agricultural pests. The first is a typical polyphagous species and represents a major threat to cotton, wheat, corn, tomato, and tobacco, the second just attacks several plant species in Solanaceae such as pepper and tobacco. The two species have different host-plant ranges, but they are very similar in other aspects [1], [2]. Interbreeding has been successfully obtained in the laboratory, in some cases with fertile hybrids [3], [4].

Odorant-binding proteins are small soluble proteins abundantly expressed in the lymph of chemosensilla [5]–[7]. Regarded for many years as passive carriers for odorant molecules to the membrane-bound olfactory receptors, now they are being recognised as the proteins responsible for detecting and discriminating the different olfactory messages. The first evidence was the demonstration that an OBP of *Drosophila*, LUSH, is required for perception of the male pheromone, vaccenyl acetate [8]. Further work showed that the same protein is able to bind and activate an olfactory receptor even in the absence of the pheromone, provided that it assumes the correct conformation [9]. At the same time, it was shown that switching two genes encoding OBPs in two species of *Drosophila* had the effect of switching their behaviour towards some fatty acids [10]. In the moth *Antheraea polyphemus*, a very elegant work has shown that the response of olfactory receptors to pheromones increases by three order of magnitude and becomes much more specific in the presence of the appropriate Pheromone-binding Protein (PBP) [11]. Finally, a recent study analysed the response to several odorants of 17 strains of *Drosophila*, each deficient in one specific OBP [12]. The results clearly show that in each strain the olfactory response was modified in different specific ways, in some cases finely tuned to one or two odorants. These data, not only provide strong evidence that OBPs are involved in odour discrimination, but also indicate that a combinatorial code is utilised by insects to recognise olfactory stimuli.

Another interesting aspect of OBP research, that is now rapidly developing, is the study of those members that are found in non-sensory organs. Particularly interesting is the occurrence of OBPs (as well as Chemosensory Proteins, CSPs, the other class of binding proteins involved in chemoreception) in structures where semiochemicals are delivered, such as pheromone glands and reproductive organs.

In the mosquito *Aedes aegypti*, OBP22 is expressed in antennae and in male reproductive organs, and is transferred to females during mating [13], [14]. In the cabbage moth *Mamestra brassicae*, a CSP has been reported in pheromone glands [15] and in the silk moth *B. mori*, a proteomic study revealed the presence of several CSPs in pheromone glands [16]. In the honey bee, *Apis mellifera*, the mandibular glands, known to be the site of synthesis and delivery of several pheromones, expressed a variety of OBPs in a caste and age dependent fashion [17].

Several OBPs have been described in *Helicoverpa armigera*. They include three Pheromone-binding proteins (PBPs), two General Odorant-binding proteins (GOBPs), one Antennal Binding protein (ABP) and nine OBPs [18], numbered as HarmOBP1 to HarmOBP9. In addition, the sequences of three PBPs and two GOBPs of *H. assulta* are available in the database under the following acc. numbers: HassPBP1: AY864775, HassPBP2: GU170398, HassPBP3: DQ286414, HassGOBP1: AY864774, HassGOBP2: AY351670.

Here we report the identification of three additional OBPs in *H. armigera* and eight in *H. assulta* and the characterization of one of them, that we name OBP10, in both species, particularly interesting for its dual expression pattern in antennae and male reproductive organs. We also show that this protein is transferred to females during mating and is eventually found on the egg shell, where it could act as a carrier for some semiochemicals.

## Results and Discussion

### Sequencing of OBPs in *H. armigera* and *H. assulta*

**Figure 1:**
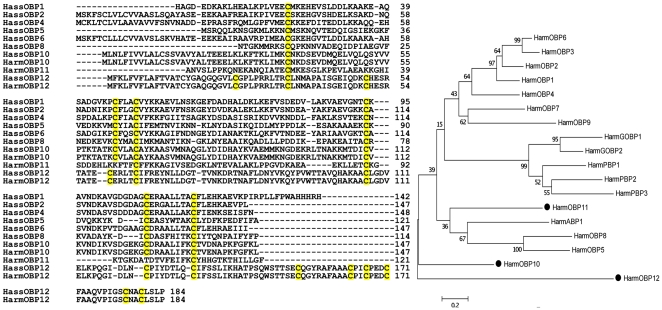
Amino acid sequences for the new OBPs identified in *H. armigera* and *H. assulta*. Differences between orthologous proteins in the two sibling species are limited to only one or few amino acid substitutions. The similarity tree on the right illustrates graphically the sequence similarity relationships between the 18 OBPs of *H. armigera* so far available. In particular, the newly identified OBP11 (a C-minus OBP with only four cysteines) and OBP12 (a C-plus OBP with 14 cysteines) fall outside the main branches of the tree. Accession numbers are as follows. HarmOBP10: JN571544; HarmOBP11: JN571545; HarmOBP12: JN571543; HassOBP1: JN571537; HassOBP2: JN571536; HassOBP4: JN571541; HassOBP5:JN571540; HassOBP6: JN571535; HassOBP8: JN571542; HassOBP10: JN571539; HassOBP12: JN571538.

Using specific primers designed on the nucleotide sequences of orthologous proteins in *H. virescens*, we obtained several amplification products encoding OBPs in both *H. armigera* and *H. assulta*. In total, we identified 10 OBPs in *H. armigera* and 8 in *H. assulta*. In the meantime, a paper was published, reporting 10 new sequences of OBPs in *H. armigera* [18]. A comparison of our sequences with those published showed that 7 of the 10 OBPs we identified in *H. armigera* had been also reported [18], although some amino acid substitutions were observed, indicating the existence of multiple forms for these proteins. Therefore, we report here on three new OBPs in *H. armigera* and 8 in *H. assulta*. Their amino acid sequences, together with their accession numbers are reported in Figure 1. We classified the three novel *H. armigera* OBPs with numbers from 10 to 12, continuing the nomenclature adopted in the literature [18]. For the sequences of *H. assulta* we assigned the same numbers reported for their orthologues in *H. armigera*. OBP10 is a “classic” OBP with the conserved six-cysteine motif, while OBP11 is a C-minus OBP (containing only 4 cysteines) and OBP12, is much longer and presents eight additional cysteines. We can confidently assign OBP11 to the group of C-minus OBPs, as the spacing between the four cysteines is rather well conserved when compared to OBPs of the same group from *Apis mellifera* [19] and *Bombyx mori* [20]. OBP12, on the other hand, is markedly different from other OBPs with additional cysteines, such as OBP47 of *Anopheles gambiae* [21], as far as the spacing between cysteines is concerned. So far, therefore, a total of 18 genes encoding OBPs of different sub-classes are available in *H. armigera*, a relatively small number when compared with the 44 genes present in the genome of *B. mori* [20]. However, we should consider that probably only a small subset of such genes might be expressed as proteins, based on the observation that in the antennae of *B. mori* only four OBPs have been identified by a proteomic study [16].

A similarity tree is reported in Figure 1 for all 18 amino acid sequences of *Harm*OBPs to show the positions of the new proteins with respect to the others. In particular, both OBP11 and OBP12 fall outside the main branches and can be considered as outliers with respect to the group of the other sequences.

A comparison between orthologous sequences in the two sibling species *H. armigera* and *H. assulta* showed only few amino acid substitutions. Similarity with the sequences of *H. virescens* was also between 70 and 95%.

### Bacterial expression and purification of HarmOBP10

For this work we decided to characterize OBP10, that, based on a PCR screening, performed on antennae, legs, proboscis, wings and pheromone glands, showed to be specifically expressed in the antennae of both sexes and both species.

OBP10 is 128 amino acid long in both species and is preceded by a signal peptide of 19 residues. It presents a “medium” C-terminus [22] constituted by 6 amino acids. In proteins of this class, the C-terminal region is not long enough to enter the binding cavity (as observed with other Lepidopteran OBPs), but plunges into the OBP core and forms an additional wall of the cavity on one side, while it is exposed to the environment on the other side. The only difference between the proteins in the two species (HarmOBP10 and HassOBP10) is a single amino acid substitution, (E119 in *H. armigera*, D119 in *H. assulta*), occurring outside the binding pocket, according to a three-dimensional model. The predicted isoelectric point for both proteins is 8.5, rather unusual if compared to the acidic values (4.5–5.5) reported for the majority of insect OBPs [7].

**Figure 2:**
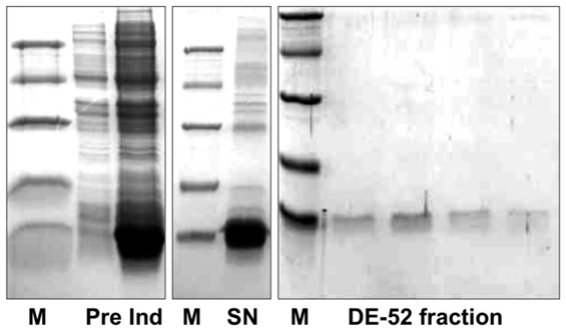
Bacterial expression and purification of HarmOBP10. The protein was obtained in high yields (about 30 mg/L of culture) as insoluble inclusion bodies and had to be denatured and renatured in order to be solubilised. Purification was accomplished by two chromatographic steps on anion-exchange resin DE-52 (Whatman). The figure reports the SDS-PAGE analysis relative to crude bacterial extracts before (**Pre**) and after (**Ind**) induction with IPTG, the crude solubilised protein (**SN**) and fractions relative to the last step of purification. Molecular weight markers (**M**) are: Bovine serum albumin (66 kDa), ovalbumin (45 kDa), carbonic anhydrase (29 kDa), trypsin inhibitor (20 kDa) and a-lactalbumin (14 kDa).

The protein was expressed in a bacterial system, using the vector pET30, and did not contain modifications with respect to the mature sequence, apart from the addition of an initial methionine. The recombinant HarmOBP10 was produced in high yields (about 30 mg/L), but as insoluble inclusion bodies. Solubilisation was accomplished by denaturation and refolding, a process that has been previously adopted for other OBPs and reported to afford the protein in fully active form [23]–[25]. Purification was performed by anion-exchange chromatography on DE-52 and QFF columns. Figure 2 reports the expression and purification of HarmOBP10. The purified protein was used to raise polyclonal antibodies in rabbit.

### Tissue and temporal expression

To investigate the expression of HarmOBP10 at the RNA level, we used PCR with specific primers at both ends of the cDNA sequence encoding the mature protein. Among antennae, proboscis, tarsi, wings and pheromone glands, we could only obtain PCR products of the expected size with the antennal samples. The gene for HarmOBP10 appeared to be equally expressed in both sexes and in both species.

Using male and female reproductive organs, we obtained strong PCR bands from the male samples of both species and very weak bands from the female samples. The amplification products from both sexes were cloned and sequenced to yield amino acid sequences very similar, but not identical to those obtained from the antennae. In particular, four clones obtained from male organs of *H. assulta* presented the same two amino acid substitutions (K9E, T89A) with respect to the antennal sequence. In *H. armigera*, instead, three clones showed different patterns of substitutions with up to 5 amino acid replacements (K9Q, V29M, Q59E, T89A, G106S).

**Figure 3:**
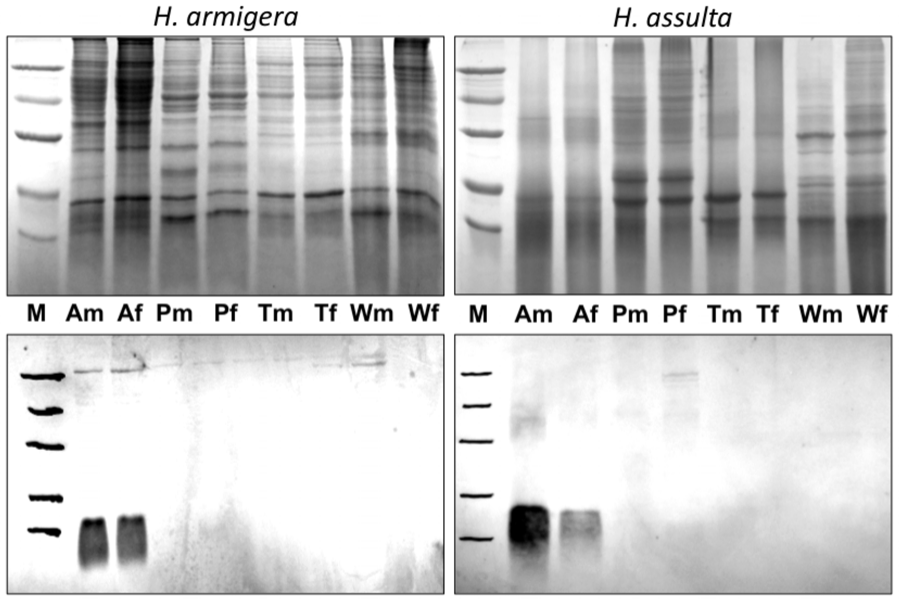
SDS-PAGE and Western blot of extracts from organs of *H. armigera* and *H. assulta* adults. Upper panels: SDS-PAGE; lower panels: Western blot. (A); antennae, (P): proboscis, (T): tarsi, (W): wings of males (m) and females (f). The expression of HarmOBP10 is limited to antennae with no significant differences between sexes or species. Molecular weight markers (**M**) are as in Figure 2.

**Figure 4:**
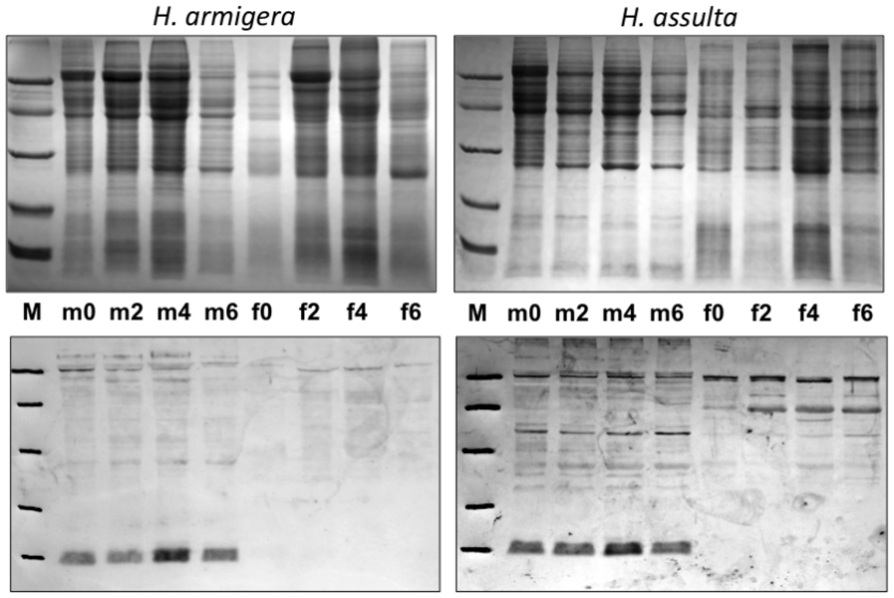
SDS-PAGE (upper panels) and Western blot analysis (lower panels) of crude extracts from reproductive organs. Reproductive organs include male accessory glands and testes, and female accessory glands and ovaries, dissected from virgin individuals of different ages. Numbers indicate days after emergence. Only male extracts of both species present an intense cross-reacting band at the expected molecular weight of OBP10. Molecular weight markers (**M**) are as in Figure 2.

**Figure 5:**
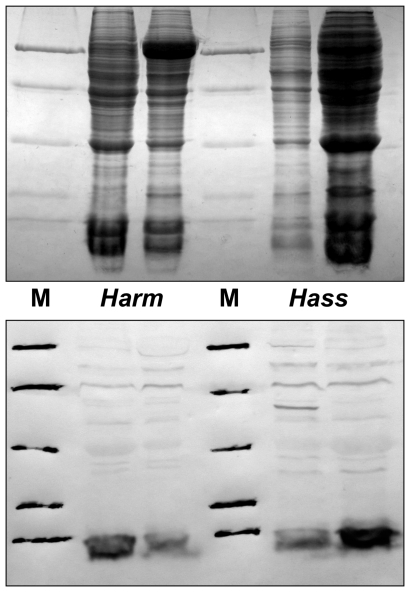
SDS-PAGE and Western blot of extracts from seminal vesicles of *H. armigera* and *H. assulta*. Upper panels: SDS-PAGE of two males for each species; lower panels: Western blot. The concentration of OBP10 in the seminal fluid is very high. Molecular weight markers (**M**) are as in Figure 2.

This expression pattern was validated at the protein level by Western blot experiments (Figure 3). Using extracts from antennae, proboscis, tarsi and wings of both sexes and both species we could only detect the protein in antennae with no significant differences between sexes or species. Besides, we also found strong expression in crude extracts of reproductive organs (Figure 4). In virgin insects, the protein is strongly expressed only in male organs of both species without significant age dependence, from emergence (day 0) to old age (day 6). Another experiment, performed with dissected seminal vesicles, revealed high concentrations of the protein, clearly indicating that OBP10 is abundant in seminal fluid (Figure 5).

**Figure 6:**
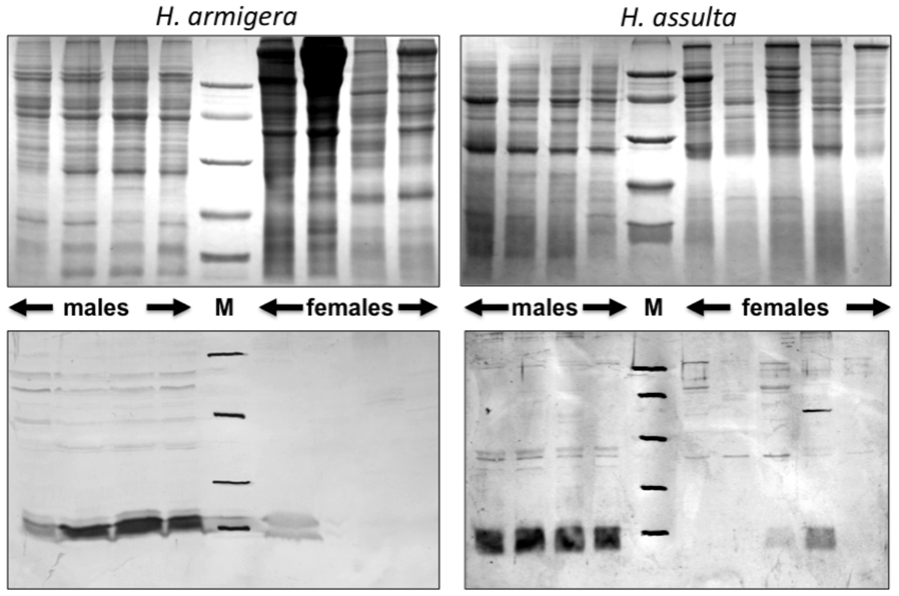
SDS-PAGE (upper panels) and Western blot analysis (lower panels) of crude extracts from reproductive organs. Reproductive organs include male accessory glands and testes, and female accessory glands and ovaries, dissected from mated individuals of 6 days. Besides males, also some female extracts contain OBP10, suggesting that the protein could have been transferred by males during mating. Molecular weight markers (**M**) are as in Figure 2.

When we performed parallel experiments with females kept overnight with males, thus being allowed to mate, the protein could also be detected in the reproductive organs of some females (Figure 6). This suggests that OBP10, whose presence in the seminal fluid has been demonstrated, is transferred from males to females during mating. The fact that only some females of the “mated” group showed the presence of OBP10 could be due to several reasons, including the possibility that mating did not occur for all the couples or was not always successful, as often has been observed when rearing insects in the laboratory.

**Figure 7:**
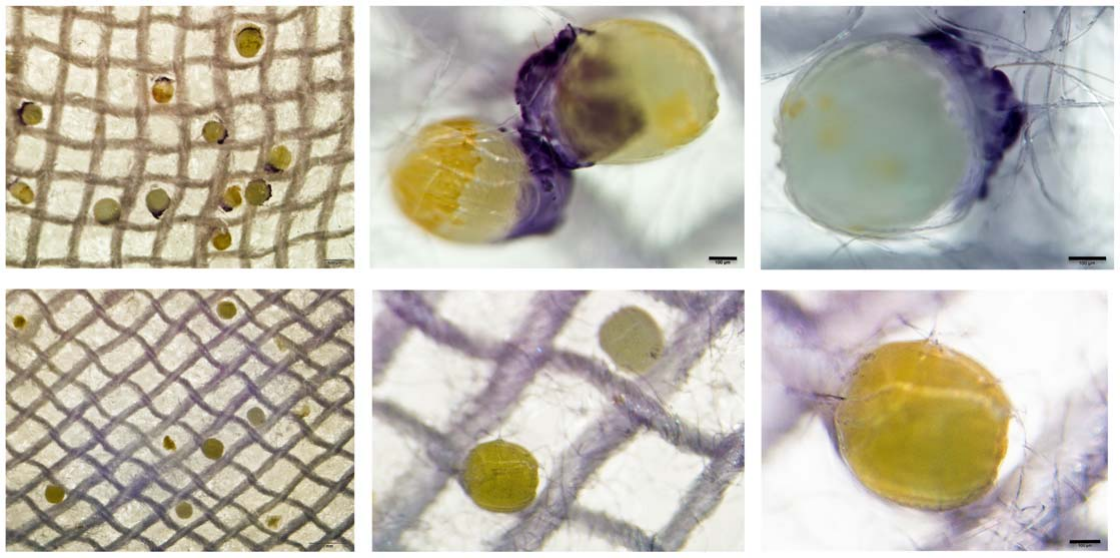
Immunostaining of OBP10 on the eggs of *H. assulta.* Eggs that had been laid on a piece of cotton were directly stained with the antiserum against OBP10, following the same protocol as for Western blot analysis. The upper panels show fertilized eggs at different magnifications. The lower panels show parallel experiments performed with unfertilized eggs, collected from females that had never been in contact with males. The heavy staining is specifically associated with fertilized eggs on the tip opposite to the micropyle. Calibration bars: 100 µm.

Then we investigated the presence of OBP10 on the eggs, performing immunostaining experiments directly on the eggs that had been laid on a piece of cotton tissue. When we used eggs from fertilised females, we detected strong reaction on all the eggs (Figure 7). The heavy staining was concentrated on a small area, on the pole opposite to the micropyle. In particular, the staining was associated with some additional material attached to the tip of the egg (Figure 7). Parallel experiments, performed on eggs laid by unfertilized females, yielded no staining (Figure 7). Moreover, the eggs, smaller and less turgid, lacked that additional external substance that was specifically stained in the eggs of fertilized females.

Having thus established that OBP10 is provided by males to females to mark fertilized eggs, we asked the question whether this protein could act as a carrier for semiochemicals. Therefore, we analysed crude dichloromethane extracts of eggs by GC/MS (data not shown). The gas-chromatographic profile contains four major peaks, identified as methyl esters of palmitic, stearic, linoleic and linolenic acids. These compounds have been reported to be oviposition deterrents in the grape moth *Lobesia botrana* [26] and, together with some fatty acids also in the corn borer *Ostrinia furnacalis* [27]. In the same paper, evidence is shown that olfaction is involved in the recognition of these deterrents by behaviour experiments performed with insect whose antennae had been amputated [27]. Moreover, the presence of fatty acids has been reported in reproductive organs and in the larval frass, as well as in the eggs of *H. armigera* [28], [29].

The presence of oviposition deterrents on the eggs is reasonable and not unexpected, as the larvae of both *H. armigera* and *H. assulta* are known to exhibit cannibalistic habits. Therefore, disseminating the eggs over a large area would increase the survival rate of larvae, and marking the fertilized eggs by the males just before oviposition should be the most efficient method to reach such result.

**Figure 8:**
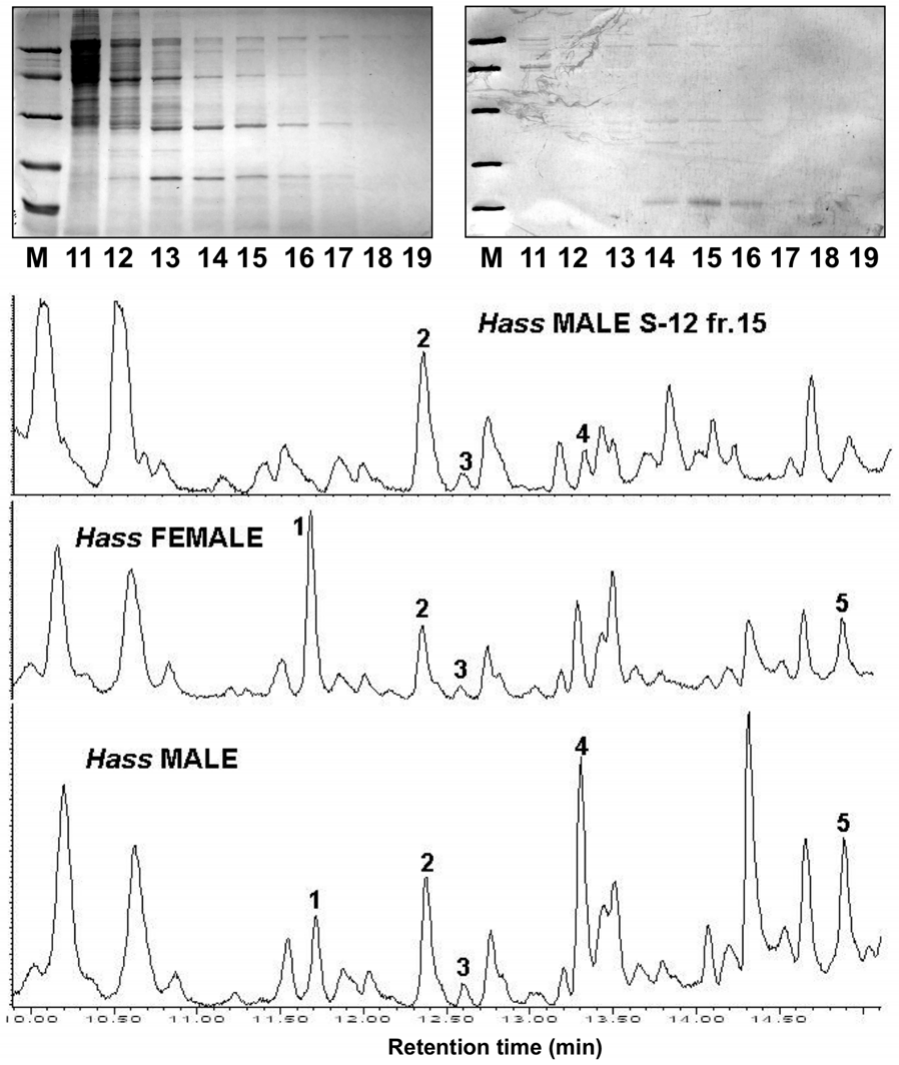
GC/MS analysis of dichloromethane extracts of reproductive organs of males and females *H. assulta.* The selected fractions (upper trace) from a Superose-12 fractionation of male reproductive organs. SDS-PAGE and Western blot analysis revealed the presence of OBP10 mainly in fraction 15. The same fraction also showed to contain 1-dodecene at higher concentration than the others. Peaks in the GC/MS profile were identified as (1) (*E*)-2-heptenal, (2) 1-dodecene, (3) dodecene (position of double bond not identified), (4) nonanal, (5) decanal. The identity of 1-dodecene has been confirmed with an authentic sample analysed in the same conditions.

Although the presence of such esters in eggs is clear and likely related to some function, binding experiments performed with OBP10 and several fatty acid esters yielded poor results, as reported later. Therefore, we analysed crude dichloromethane extracts of reproductive organs of males and females, searching for other putative semiochemicals that could be carried by OBP10. Figure 8 reports the GC/MS profiles of such extracts performed on *H. assulta* organs, while very similar results have been obtained with samples of *H. armigera* (not shown). A great number of peaks are present and several hydrocarbons, as well as medium chain aldehydes and other compounds could be recognised. In order to identify which of these chemicals could be bound to OBP10, we fractionated a crude extract from male reproductive organs of *H. assulta* on a gel filtration column and analysed each fraction by SDS-PAGE, Western blot using the antiserum against OBP10, and GC/MS after extraction with dichloromethane. The results are also reported in Figure 8 and match very closely parallel data obtained with *H. armigera* (not shown). In both species, we identified a peak eluted in our conditions at a retention time of 12.3 minutes as 1-dodecene. The intensity of this peak is higher in the fractions containing the highest concentration of OBP10, as evaluated in Western blot experiments (Figure 8), indicating this compound as a likely endogenous ligand of the protein. However, the data so far obtained do not allow to exclude that other volatiles present in the extract of male reproductive organs could represent the endogenous ligands for OBP10. The identity of 1-dodecene has been confirmed by GC/MS analysis of an authentic sample, performed in the same conditions. This peak is immediately followed by a minor one, also tentatively identified as dodecene, but for which we could not establish the position of the double bond. There is not much information in the literature regarding 1-dodecene as a semiochemical. This compound has been reported as an important component of the postpharyngeal gland in two species of ants, *Pogonomyrmex salinus* and *Messor lobognathus* [30]. Three dodecene isomers were also identified in the mandibular secretions of some meliponine worker bees [31]. As for other linear alkenes, 1-tridecene, has been reported as a male sex pheromone of the tenebrionid beetle *Parastizopus transgariepinus* [32]. This compound is secreted by the aedeagal gland, while other alkenes, such as 1-undecene and 1-heptadecene are present in the secretion of the pygidial defensive gland in the same species [33], as well as other tenebrionids [35]. Moreover, 1-dodecene has been recognised as one of the main compounds responsible for the repellence of soybean leaves towards the Lepidopteran *Trichloplusia ni* and the coleopteran *Epilachna varivestis* [34]. Based also on such data, a few patents describing products to be used as insect repellents have included 1-dodecene in their formulations [35].

At this point we decided to look more carefully for the presence of 1-dodecene in egg extracts. In fact, when we analysed extracts from fresh eggs, laid within 3–4 hours, we could detect a small peak corresponding to 1-dodecene, which was however completely absent from eggs that had been collected after 24 hours (data not shown).

From a chemical point of view, the short life of this compound in the environment might be due to the instability of the terminal double bond, as well as to its volatility. The ecological reason for the short effect of this putative repellent is probably related to the fact that its permanence for a long time in the environment would unnecessarily inhibit oviposition to a large extent.

### Ligand-binding experiments

**Figure 9:**
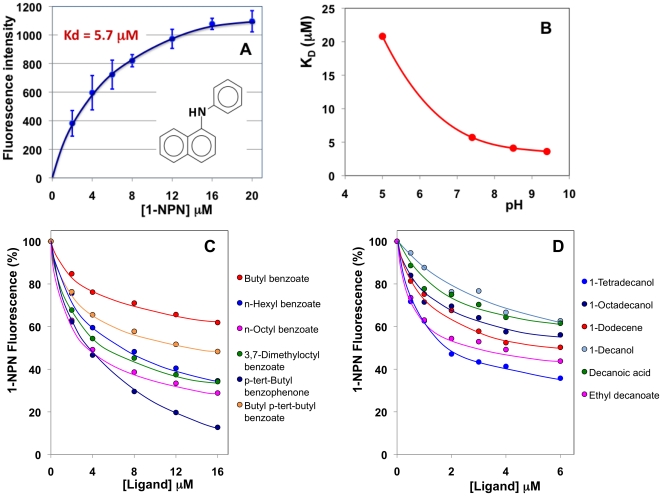
Binding of 1-NPN and selected ligands to HarmOBP10. (**A**) Affinity of 1-NPN to the recombinant protein. 2 µM solution of the protein in Tris was titrated with 1 mM solution of 1-NPN in methanol to final concentrations of 2–16 mM. The data, averages of three replicates, were analysed using Prism software and indicated the presence of a single binding site with a dissociation constant of 5.7 µM. (**B**) Effect of pH on the dissociation constant of the complex HarmOBP10/1-NPN. Best affinities are measured at high pH values. (**C**) and (**D**). Examples of competition binding assays. In each experiment a mixture of the protein and 1-NPN in Tris, both at the concentration of 2 µM, was titrated with the competing ligand to final concentrations of 2–16 µM. Fluorescence intensities are reported as percent of the values in the absence of competitor. The full set of data relative to all the ligands testes is reported in Table 1.

In order to investigate the specificity of the binding pocket in OBP10 and verify its compatibility with a carrier for 1-dodecene, we performed ligand-binding experiments along with well established protocols for other insect OBPs. We first measured the affinity to the fluorescent probe 1-NPN, that binds OBP10 with an isotherm indicating a single binding site with a dissociation constant of 5.7 µM at pH 7.4 (Figure 9A). We then measured the affinity to the same probe across a range of pH from 5 to 9.4. The curve, reported in Figure 9B, indicates a marked loss of affinity in acidic conditions, but similar and even better dissociation constants in basic buffers. In this respect, we can observe that HarmOBP10, unlike most insect OBPs, presents a rather high calculated isoelectric point of 8.5. Affinities of other ligands were then evaluated in competitive binding experiments, where we measured the ability of each compound to displace the fluorescent probe from the complex. Selected curves are reported in Figure 9C and 9D, while the binding parameters for all the compounds tested are listed in Table 1.

**Table 1:** Binding affinities of ligands to recombinant HarmOBP10.

Ligand	max conc	% at max	IC_50_	K_D_
*Terpenoids*				
Geraniol	16	69		
Citralva	16	60		
Citral	16	66		
Hydroxycitronellal	16	48	13.5	10.5
Hydroxycitronellic acid	16	50	16	12.4
3,7-Dimethyloctyl acetate	16	59		
Farnesol	6	54	7	5.4
*Aromatic compounds*				
n-Butyl benzoate	16	62		
n-Hexyl benzoate	16	35	7.5	5.8
n-Octyl benzoate	16	29	4	3.1
3,7-Dimethyloctyl benzoate	16	34	6	4.7
p-tert-Butylbenzophenone	16	13	3.3	2.6
Butyl p-tert-butylbenzoate	16	48	14	10.9
Cinnamic acid	16	74		
Methyl cinnamate	16	>100		
n-Butyl cinnamate	16	42	12.5	9.7
a-Methoxycinnamaldehyde	16	37	12.5	9.7
a-Amylcinnamaldehyde	16	13	3.5	2.7
Homovanillic acid	16	66		
Eugenol	16	80		
Coniferyl aldehyde	16	30	7.5	5.8
2-Phenylethyl acetate	16	77		
4-Phenylbutyric acid	16	73		
4-Phenylanisole	16	65		
Cyclamen aldehyde	16	>100		
p-Isopropylbenzaldehyde	16	77		
2-Methylnaphthalene	16	75		
*Linear compounds*				
1-Dodecene	6	50	6	4.7
1-Decanol	6	63		
1-Dodecanthiol	6	62		
1-Tridecanol	6	85		
1-Tetradecanol	6	36	1.8	1.4
1-Hexadecanol	6	55		
1-Octadecanol	6	56		
Dodecyl acetate	16	>100		
Ethyl decanoate	6	44	5.5	4.3
Ethyl laurate	6	70		
Ethyl myristate	6	73		
Ethyl palmitate	6	69		
Decanoic acid	6	62		
Linoleic acid	6	56		
Nonanal	16	>100		
Decanal	16	>100		
Dodecanal	6	73		
2-Dodecanone	16	51	17	13.2
2-Tridecanone	16	60		
(*Z*)-11-Hexadecenal	1.6	76		
(Z)-9-Hexadecenal	1.6	73		
(Z)-11-Hexadecen-1-ol	1.6	74		
(Z)-9-Hexadecen-1-ol	1.6	65		

The maximum concentration used for the binding assays and the percent of fluorescence measured at that concentration are reported, together with the concentration of ligand halving the initial fluorescence value (IC_50_) and the calculated dissociation constants (K_D_).

It is clear that the best ligands are relatively large molecules with one or two aromatic rings. In particular, the measurements with the series of alkyl benzoates indicate the ideal size for the best ligands. While butyl benzoate (10 carbon atoms) is a poor ligand, the hexyl, octyl and dimethyloctyl derivatives (12, 14 and 16 carbons, respectively) are all rather good ligands, octyl benzoate being the best. Even better ligands are *p*-*tert*-butylbenzophenone and α-pentylcinnamaldehyde. The series of aliphatic primary alcohols shows a maximum of affinity for 1-tetradecanol, while shorter and longer linear alcohols are much poorer ligands. Most interestingly, 1-dodecene proved to be a rather good ligand, with a calculated dissociation constant of 4.7 µM.

**Figure 10:**
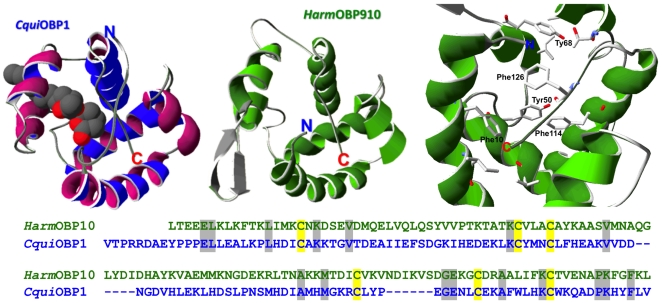
Three-dimensional folding of CquiOBP1 (acc. No. 3OGN) of *Culex quinquefasciatus* and model of HarmOBP10. The model of HarmOBP10 was based on the structure of CquiOBP1. The two proteins share only 19% of their residues, but the overall folding appears rather similar, apart from two extra loops in HarmOBP10. The amino acid residues lining the binding pockets in the two proteins, however, appear rather different. HarmOBP10 contains a relatively large number of aromatic groups, Phe10, Tyr50, Tyr68, Phe114 and Phe126, as well as other hydrophobic residues, such as Ile14, Val45 and Ile64. Of these only Phe126 is also present in CquiOBP1,while Tyr50 is replaced in the mosquito protein by a Phe. Such differences can be related to the different structures of the ligands in the two proteins, a cyclic terpenoid molecule for CquiOBP1 and linear ligands for our OBP10. Amino- and carboxy termini are indicated as N and C, respectively. Models have been generated using the programme Swiss-Model [42]–[44]. Models were displayed using the SwissPdb Viewer programme “Deep-View” [42] (http://www.expasy.org/spdbv/).

A model of HarmOBP10 (Figure 10) was built on the structure of *Culex quinquefasciatus* OBP1 as a template, a protein that has been reported to be a carrier for an oviposition pheromone [36]–[39]. The two proteins only share 19% of their residue, but the overall folding is rather similar. However, marked differences can be observed in their binding sites. The OBP10 of *H. armigera* contains an exceptionally high number of aromatic residues in its pocket, namely Phe10, Tyr50, Tyr68, Phe114 and Phe126, in addition to other hydrophobic residues, such as Ile14, Val45 and Ile64 (Figure 10). It is reasonable to imagine that the double bond of 1-dodecene might establish interactions with one of the aromatic rings, while the rest of the chain remains buried in the hydrophobic pocket. The good affinity of linear benzoates, as well as the other aromatic compounds could also easily be justified, given the presence of several aromatic residues in the binding cavity. On the other hand, the structure of 1-dodecene can be easily superimposed to those of hexyl and octyl benzoates, matching the vinyl group of the former molecules with the benzene ring of the esters.

The fact that 1-dodecene is not the best ligand for OBP10 should not invalidate the idea that the protein could be a carrier for this unsaturated hydrocarbon. In fact, it has been often observed that some synthetic compounds act as better ligands than the natural ones. One clear example is that of the alarm pheromone for aphids, β-farnesene, that is not the best ligand for OBP3, although other elements of evidence strongly suggest that such protein in involved in the perception of this semiochemical [40]. On the other hand, it is known that the binding pocket of insect OBPs is rather plastic and can even increase substantially its volume to accommodate different ligands [22].

In conclusion, the likely functions of this proteins are (1) to carry an oviposition deterrent for marking fertilized eggs (therefore their presence only in the seminal fluid) in order to reduce cannibalistic habits of this and related species; (2) to perceive the same semiochemicals through the antennae immediately after laying the first egg, thus prompting the female to move to other locations for continuing its egg laying process.

## Materials and Methods

### Insects

*H. armigera* and *H. assulta* were collected as larvae from Zhengzhou, Henan province of China. The larvae were reared in the laboratory on artificial diet, the main components of which were wheat germ and tomato paste. Rearing took place at a temperature of 27±1°C with a photoperiod of 16h∶8h, L∶D. Pupae were sexed and males and females were put into separate cages for eclosion. When eclosed, adults were given 10% honey solution.

### Reagents

All enzymes were from New England Biolabs. Oligonucleotides were custom synthesized at Augct Biotechnology, Beijing, China. All other chemicals were purchased from Sigma-Aldrich and were of reagent grade, except selected compounds used in binding assays, which were prepared along with conventional synthetic routes.

### RNA extraction and cDNA synthesis

Total RNA was extracted from TRI® Reagent (Invitrogen), following the manufacturer's protocol. cDNA was prepared from total RNA by reverse transcription, using 200 units of M-MLV Reverse Transcriptase (Promega) and 0.5 µg of an oligo-dT primer in a 50 µl total volume. The mixture also contained 0.5 mM of each dNTP (TaKaRa), 75 mM KCl, 3 mM MgCl_2_, 10 mM DTT and 0.1 mg/ml BSA in 50 mM Tris-HCl, pH 8.3. The reaction mixture was incubated at 42°C for 60 min and the product was directly used for PCR amplification or stored at −20°C.

### Polymerase chain reaction

Aliquots of 1 µL of crude cDNA were amplified in a Bio-Rad Gene CyclerTM thermocycler, using 2.5 units of Thermus aquaticus DNA polymerase (TaKaRa), 1 mM of each dNTP (TaKaRa), 1 µM of each PCR primer, 50 mM KCl, 2.5 mM MgCl_2_ and 0.1 mg/ml BSA in 10 mM Tris-HCl, pH 8.3, containing 0.1% v/v Triton X-100. For obtaining the sequences of OBPs in our species, we used specific primers at both ends corresponding to the first 17 bases and the last 18 bases of the full-length sequence of orthologous gene of the closely related species *Heliothis virescens*.

For the recombinant HarmOBP10, at the 5′ end we used a specific primer encoding the first six amino acids of the mature protein, preceded by an Nde I restriction site. At the 3′ end, the primer contained the sequence encoding the last six amino acids, followed by a stop codon and an Eco RI restriction site. After a first denaturation step at 95°C for 5 min., we performed 35 amplification cycles (1 min. at 95°C, 30 sec. at 50°C, 1 min. at 72°C) followed by a final step of 10 min. at 72°C. In all experiments we obtained amplification products of 350–500 bp, in agreement with the expected sizes.

### Cloning and sequencing

The crude PCR products were ligated into a pGEM (Promega) vector without further purification, using a 1∶5 (plasmid∶insert) molar ratio and incubating the mixture overnight, at room temperature. After transformation of *E. coli* Top10 competent cells with the ligation products, positive colonies were selected by PCR using the plasmid's primers SP6 and T7, grown in LB/ampicillin medium and custom sequenced at Augct Biotechnology, Beijing, China.

### Cloning in expression vectors

pGEM plasmid containing the sequence encoding the mature protein, flanked by the two restriction sites, was digested with Nde I and Eco RI restriction enzymes for two hours at 37°C and the digestion product was separated on agarose gel. The obtained fragments was purified from gel using TaKaRa MiniBest Plasmid Purification Kit (TaKaRa) and ligated into the expression vector pET30b (Novagen, Darmstadt, Germany), previously linearized with the same enzymes. The resulting plasmid was sequenced and shown to encode the mature protein.

### Preparation of the protein

For expression of the recombinant HarmOBP10, pET-30b vector containing the sequence encoding the mature protein was used to transform BL21 *E. coli* cells. Protein expression was induced by addition of IPTG to a final concentration of 0.4 mM when the culture had reached a value of O.D.600 = 0.8. Cells were grown for further 2 hours at 37°C, then harvested by centrifugation and sonicated. After centrifugation, OBP10 was present as inclusion bodies. To solubilise the protein, the pellet from 1 L of culture was dissolved in 10 mL of 8 M urea, 1 mM DTT in 50 mM Tris buffer, pH 7.4, then diluted to 100 mL with Tris buffer and dialysed three times against Tris buffer. Purification of the protein was accomplished by combinations of chromatographic steps on anion exchange resins, such as DE-52 (Whatman) and QFF, along with standard protocols previously adopted for other odorant-binding proteins.

### Preparation of the antiserum

An antiserum against HarmOBP10 was obtained by injecting a rabbit subcutaneously and intramuscularly with 300 µg of recombinant protein, followed by three additional injections of 150 µg after 10 days each time. The protein was emulsified with an equal volume of Freund's complete adjuvant for the first injection and incomplete adjuvant for further injections. The rabbit was bled one week after the last injection and the serum was used without further purification. The rabbit was housed in a large cage, at constant temperature, and all operations were performed according to ethical guidelines to minimize pain and discomfort to the animal.

### Western blot analysis

After electrophoretic separation under denaturing conditions (14% SDS-PAGE), duplicate gels were stained with 0.1% Coomassie blue R250 in 10% acetic acid, 20% ethanol or electroblotted on Trans-Blot nitrocellulose membrane (Bio-Rad Lab) by the procedure of Kyhse-Andersen [41]. After treatment with 2% powdered skimmed milk/Tris overnight, the membrane was incubated with the crude antiserum against the protein at a dilution of 1∶500 (2 h), then with goat anti-(rabbit IgG) horseradish peroxidase conjugate (dilution 1∶1000; 1 h). Immunoreacting bands were detected by treatment with 4-chloro-1-naphthol and hydrogen peroxide.

### Direct immunostaining of eggs

Eggs of both species, laid on a piece of cotton tissue were stained with the antiserum against HarmOBP10 without removing them from the tissue and following the same protocol used for Western blot experiments.

### Fluorescence measurements

Emission fluorescence spectra were recorded on a Hitachi F-4500 at 25°C in a right angle configuration, with a 1 cm light path quartz cuvette and 5 nm slits for both excitation and emission. The protein was dissolved in 50 mM Tris-HCl buffer, pH 7.4, while ligands were added as 1 mM methanol solutions.

### Fluorescence binding assays

To measure the affinity of the fluorescent ligand 1-NPN to HarmOBP10, a 2 µM solution of the protein in 50 mM Tris-HCl, pH 7.4, was titrated with aliquots of 1 mM ligand in methanol to final concentrations of 2–16 µM. The probe was excited at 337 nm and emission spectra were recorded between 380 and 450 nm. The affinity of other ligands was measured in competitive binding assays, where a solution of the protein and 1-NPN, both at the concentration of 2 µM was titrated with 1 mM methanol solutions of each competitor over concentration ranges of 0.5–1.6 µM, 0.5–6 µM or 2–16 µM, depending on the ligand. Dissociation constant for 1-NPN and the stoichiometry of binding was obtained processing the data with Prism software. Dissociation constants of the competitors were calculated from the corresponding IC_50_ values (concentrations of ligands halving the initial fluorescence value of 1-NPN), using the equation: K_D_ = [IC_50_]/1+[1-NPN]/K_1-NPN_, [1-NPN] being the free concentration of 1-NPN and K_1-NPN_ being the dissociation constant of the complex protein/1-NPN.

### Gas-chromatography/mass spectrometry analysis

To identify organic compounds present in the reproductive organs, the homogenate from three organs in 2 mL of Tris buffer was extracted with 2 mL of dichloromethane. In the case of eggs, about 60 eggs were directly extracted with 1 mL of dichloromethane by incubating them in the solvent for one day at room temperature. To identify the endogenous ligands of OBP10, a crude extract of male reproductive organs was fractionated on a gel filtration Superose-12 (1×30 cm) column. The fractions were analysed by SDS-PAGE and Western blot, as well as extracted with dichloromethane. In all cases, organic phases were concentrated to about 20 µL and 2 µL used for GC/MS analysis. The samples were analysed with an Agilent Technologies 5975 C MS (Agilent) coupled to an Agilent Technologies 7890 A GC (Agilent) equipped with polar DB-WAX fused silica column (30 m×0.25 mm ID, 0.25 µm film, J&W Scientific Inc., Folsom, CA, USA) or nonpolar DB-5 fused silica column (30 m×0.25 mm ID, 0.25 µm film, J&W Scientific Inc., Folsom, CA, USA). The temperature programme was as follows: 50°C for 5 min, then 10°C/min to 220°C and held at 220°C for 15 min, finally held at 250°C. Windows NIST08 Spectral Search Program (Version 1.7) software was used for data analysis. Injections were made in the split less mode. Helium was used as the carrier gas (1.0 ml/min). For electron impact (EI) mass spectra, the ionization voltage was 70 eV and the temperatures of the ion source and of the interface were 230°C and 280°C, respectively. The emission current was 34.6 µA. Compounds were identified using the mass spectral database.

### Molecular modelling

A three-dimensional model of HarmOBP10 was generated using the on-line programme SWISS MODEL [42]–[44]. The structure of OBP1 of *Culex quinquefasciatus*, acc. No. 3OGN, was used as a template (identity between the two proteins: 19%). Models were displayed using the SwissPdb Viewer programme “Deep-View” [42] (http://www.expasy.org/spdbv/).
